# Study of antimicrobial resistance due to extended spectrum beta-lactamase-producing *Escherichia coli* in healthy broilers of Jabalpur

**DOI:** 10.14202/vetworld.2016.1259-1263

**Published:** 2016-11-16

**Authors:** Arpita Shrivastav, R. K. Sharma, Y. P. Sahni, Neeraj Shrivastav, Vidhi Gautam, Sachin Jain

**Affiliations:** 1 Department of Veterinary Pharmacology & Toxicology, College of Veterinary Science & Animal Husbandry, Nanaji Deshmukh Veterinary Science University, Jabalpur, Madhya Pradesh, India; 2 Director Research Services, Nanaji Deshmukh Veterinary Science University, Jabalpur, Madhya Pradesh, India; 3 Department of Veterinary Microbiology, College of Veterinary Science & Animal Husbandry, Nanaji Deshmukh Veterinary Science University, Jabalpur, Madhya Pradesh, India

**Keywords:** cecal swab, *Escherichia coli*, extended spectrum beta-lactamase, healthy broilers, Jabalpur

## Abstract

**Aim:**

To study the prevalence of antimicrobial resistance due to extended spectrum beta-lactamase (ESBL)-producing *Escherichia coli* in samples collected from the ceca of healthy broilers of poultry sale outlets (PSOs) Jabalpur.

**Materials and Methods:**

A total of 400 cecal swab samples were taken randomly from freshly slaughtered poultry of 39 PSOs located at four different zones or areas of Jabalpur and were screened for the presence of ESBL-producing *E. coli* using standard methods. Further they were characterized phenotypically by standard methods.

**Results:**

All the 400 samples were screened for *E. coli* producing ESBL enzyme. Among the samples positive for *E. coli* 135 were positive for ESBL *E. coli* giving an overall prevalence of 33.5%.

**Conclusion:**

This study related to the prevalence of ESBL-producing *E. coli* in healthy broilers in Jabalpur is indicative of antibiotic resistance prevalent in the healthy birds which are used for human consumption as well. It also signifies resistance prevalent against beta-lactam antibiotics including third and fourth generations of cephalosporins.

## Introduction

Antimicrobial resistance, within a large range of infectious agents, is a rising health risk of broad concern to countries and multiple sectors. It not only menaces the effective prevention and treatment of an ever-increasing range of infections but also results in reduced efficacy of antibacterial drugs. In intensively reared poultry, antibiotics are administered to whole flocks rather than individual animals. In addition to this poultry farmer also use low doses of antibiotics as growth-promoting substances, which result in the high antibiotic selection pressure for resistance with relatively high proportion of resistant bacteria in poultry fecal flora.

Most resistant phenotypes present in animal populations are present in *Escherichia coli*, therefore commensal *E. coli* can be used as indicators of the Gram-negative species. During the passage through the intestine, these bacteria may transfer their resistance genes to host-adapted bacteria or to pathogens. All animals generally carry such indicator bacteria this is why trends in the occurrence of resistance, can be studied more accurately in indicator bacteria [1].

Beta-lactams (penicillins, cephalosporins, carbapenems, and monobactams) constitute the therapy of choice for some well-established practices and infections in veterinary medicine [2]. The third generation of cephalosporins has been associated with the emergence of beta-lactamases mediated bacterial resistance, which subsequently led to the development of extended spectrum beta-lactamase (ESBL)-producing bacteria.

ESBLs have been defined as plasmid-encoded enzymes found in the Enterobacteriaceae [3], frequently in *E. coli* and *Klebsiella pneumoniae*, that confer resistance to a variety of beta-lactam antibiotics by catalyzing the hydrolysis of the beta-lactam ring of antibiotic specially oxyimino-cephalosporins, which can be inhibited by beta-lactamase inhibitors [4].

ESBL-producing organisms are frequently co- or multi-resistant, exhibiting resistance to other antimicrobial classes such as fluoroquinolones, aminoglycosides, and trimethoprim-sulfamethoxazole due to associated resistance mechanisms, which may be either chromosomally- or plasmid-encoded [5,4].

During the last two decades, ESBL-producing Gram-negative bacilli have emerged as a major problem mainly due to the clonal expansion of producer organisms, the horizontal transfer of ESBL genes on plasmids [6]. Apart from therapy and prophylaxis, antibiotics are consumed to increase growth and feed efficiencies. There was a limited number of drugs sensitivity for these bacteria only and drug of choice is imipenem, followed by amikacin in injectable form. However, most probably in the near future, if this irrational use is not stopped, infection with that Gram-negative bacteria increase the rate of resistant to drugs that are now sensitive, resulting increase morbidity and mortality. Looking into the severity of the problem present study was undertaken for the prevalence and characterization of ESBL-producing *E. coli* in healthy broilers.

## Materials and Methods

### Ethical approval

No ethical approval was required as no live animals were used in this study. However, samples were collected as per standard sample collection methods following all aseptic precautions.

### Study site

The study was conducted at Department of Veterinary Pharmacology and Toxicology, College of Veterinary Science and Animal Husbandry, Jabalpur, during January 2015 to January 2016.

### Sample collection

A total of 400 cecal swab samples were collected randomly from 38 poultry sale outlets (PSOs) located at the various parts of Jabalpur. Sample collection area was divided into four zones east, west north and south and five areas were selected randomly in each zone area (Table-1). Samples were taken from the freshly slaughtered healthy broilers in an ice pack and taken to the lab. The properly labeled interlocked polythene bags containing the ceca were brought to the laboratory of the Department of Pharmacology and Toxicology, College of Veterinary Science and Animal Husbandry, Jabalpur for further study.

**Table 1:** List of different sample collection areas of Jabalpur.

Name of the area	Number of samples
North Zone 1	12
North Zone 2	31
North Zone 3	12
North Zone 4	10
North Zone 5	58
South Zone 1	8
South Zone 2	13
South Zone 3	17
South Zone 4	34
South Zone 5	5
East Zone 1	11
East Zone 2	17
East Zone 3	23
East Zone 4	17
East Zone 5	10
West Zone 1	11
West Zone 2	5
West Zone 3	95
West Zone 4	7
West Zone 5	4
Total samples	400

### Sample processing

Taking all, the standard aseptic measures directly ceacal material were collected by incising the intact ceca with the help of sterile B.P.blade, later sterile swab swirled around and immediately transferred into the enrichment medium containing buffered peptone water 25 ml/5 g of sample for increasing the sensitivity and clonal expansion of the ESBL producing *E. coli*. Further, it was transferred into the M.H. broth supplemented with cefotaximes and cefpodoxime (2 µg/ml) and aztreonam (4 µg/ml) for the selective enrichment. Overnight enriched samples were streaked into the tryptone bile X-glucuronic agar plate supplemented with cefotaxime and aztreonam in the above-mentioned concentration for further selection of desired organisms. For further confirmation, the phenotypic characterization of ESBL producing *E. coli* was undertaken using standard methods combined disc diffusion test (CDDT) method, double disc synergy test method (DDST), and Ezy MIC strip method.

## Results and Discussion

This study revealed the presence of ESBL producing *E. coli* in the healthy broilers of Jabalpur. Out of the total 400 cecal swab samples screened, 135 samples were found to be positive for ESBL giving an isolation prevalence percent of 33.5% as given in Table-1. Previously, different workers have reported the prevalence of similar ESBL-producing *E. coli* in healthy boilers.

In the present investigation initial screening in the buffered peptone water and M.H. broth and later in chromogenic medium specific for *E. coli* enriched with cefotaxime (2 µg/ml), cefpodoxime (2 µg/ml), and aztreonam (4 µg/ml) shown 135 samples positive out of 400 samples and here resistance to cefotaxime ceftazidime, and cefpodoxime whereas susceptibility to cefoxitin further confirms the presence of ESBL *E.coli*. In the present study entire sample collection area was divided into four zones which was North, South, East and West zone (Table-1). Broadly five areas were taken in each zone as mentioned in the Table-2. Highest prevalence was seen in the West zone (48.36%), whereas lowest prevalence was seen in the South zone (25.97%). In North and East zone the prevalence was in the range of 28.45% and 26.92% (Table-2). Different poultry sale outlets were further included in these zones which showed a wide variation in the results (Table-3). Perusal of the results revealed that at some areas there was 100% prevalence of ESBL *E. coli* isolates, whereas other areas exhibited lower range of prevalence (0-30%). Our findings simulate with the results obtained by Hasan *et al*. [7] in Bangladesh and adjoining areas of India where they observed an overall prevalence of ESBL-producers as 30% in poultry and domestic birds, 27% in wild birds and 59% prevalence was seen in hospitals and community people, they further concluded that ESBL-producing bacterial species diversity was highest in poultry and humans were the best ESBL carriers.

**Table 2:** Percent prevalence of ESBL *E. coli* from different collection areas of Jabalpur.

Name of the area	Number of samples	Positive samples	Negative samples	Percent prevalence
North Zone 1	12	7	5	58.3
North Zone 2	31	2	29	6.5
North Zone 3	12	8	4	66.7
North Zone 4	10	9	1	90.0
North Zone 5	58	9	49	15.5
South Zone 1	8	8	0	100.0
South Zone 2	13	4	9	30.8
South Zone 3	17	7	10	41.2
South Zone 4	34	0	34	0.0
South Zone 5	5	1	4	20.0
East Zone 1	11	1	10	9.1
East Zone 2	17	7	10	41.2
East Zone 3	23	5	18	21.7
East Zone 4	17	0	17	0.0
East Zone 5	10	8	2	80.0
West Zone 1	11	6	5	54.5
West Zone 2	5	0	5	0.0
West Zone 3	95	53	42	55.8
West Zone 4	7	0	7	0.0
West Zone 5	4	0	4	0.0
Total samples	400	135	265	

ESBL=Extended spectrum beta-lactamase, *E. coli*=*Escherichia coli*

**Table 3:** Percent prevalence of ESBL *E. coli* in different PSOs of Jabalpur.

Zone	Name of PSO	Number of samples collected	Number of positive samples	Number of negative samples	Percent prevalence
North	PSO 1	12	7	5	58.3
	PSO 2	1	1	0	100.0
	PSO 3	9	8	1	88.8
	PSO 4	8	0	8	0.00
	PSO 5	16	2	14	12.5
	PSO 6	7	3	4	42.85
	PSO 7	28	1	27	3.57
	PSO 8	29	8	21	27.58
	PSO 9	7	2	1	28.57
	PSO 10	3	3	0	100.0
	PSO 11	2	0	2	0.00
South	PSO 1	8	8	0	100.0
	PSO 2	10	2	2	20
	PSO 3	3	0	3	0.00
	PSO 4	5	0	5	0.00
	PSO 5	6	3	3	50.0
	PSO 6	11	2	9	18.1
	PSO 7	34	0	34	0.00
East	PSO 1	17	0	17	0.00
	PSO 2	10	5	5	50.0
	PSO 3	4	0	4	0.00
	PSO 4	4	4	0	100.0
	PSO 5	9	3	6	33.3
	PSO 6	6	5	1	83.3
	PSO 7	3	1	2	33.3
	PSO 8	14	0	14	0.00
	PSO 9	12	2	10	16.6
West	PSO 1	4	0	4	0.00
	PSO 2	7	6	1	85.7
	PSO 3	5	2	3	40
	PSO 4	15	2	13	13.3
	PSO 5	10	8	2	80.0
	PSO 6	5	2	3	40.0
	PSO 7	5	0	5	0.00
	PSO 8	39	33	6	84.61
	PSO 9	19	11	8	57.89
	PSO 10	2	1	1	50.0
	PSO 11	7	0	7	0.00
	PSO 12	4	0	4	0.00

PSO=Poultry sale outlet, ESBL=Extended spectrum beta-lactamase, *E. coli*=*Escherichia coli*

A wide range of prevalence from 0% to 100% in the present investigation also revealed that occurrence of this varied range of resistant isolates does not correlates only with direct use of antibiotics, but even other species of birds and humans, can carry antibiotic resistance traits, including ESBL-producers and bring resistance in broiler birds, as these ESBL-producers have already spilled over into the environment [8]. A study performed by van den Bogaard *et al*. [9] indicated that transmission of resistant clones and resistance plasmids of *E. coli* from poultry to humans commonly occurs. In this study, the prevalence of resistance in fecal *E. coli* in broilers and turkeys was analyzed, and the highest prevalence of resistance was detected in turkey samples, closely followed by those from broilers.

In a study conducted in 14 different chicken farms in Henan Province in China 51 nonreplicate ESBL-producing *E. coli* were isolated. 31 of the 51 isolates were positive for an ESBL phenotype and 29 of these isolates carried one or more *Bla* genes [10]. Another study on Belgian broiler farms, concluded that risk factors associated with the occurrence of ESBL-producing *E. coli* besides the usage of any particular antimicrobial like cephalosporins, also included generic antimicrobial use [3,11] the cleanliness of the environment, the lack of acidification of drinking water, the application of more than three feed changes during the production cycle, the breed and the litter material that is used [12,13]. The aforesaid findings add another aspect to our study that, in spite of obtaining birds from same sources prevalence varied to great extent because at the level of farm management, ESBL producing bacteria may enter and proliferate in a farm through the stocking of new animals, exposure to contaminated air, through water or feed, insect or rodent vectors, human-to-animal and animal-to-animal transmission. Moreover, chemicals used in animal production - such as antiseptics, disinfectants, and metals - could play a role in the appearance of such resistant isolates [3,14-16].

In the phenotypic characterization by the CDDT method, out of 135 samples, all the samples were found to be positive and none of the samples were negative calculating a percent isolation of 100%. In DDST method out of, 135 samples screened 115 samples shown the positive results. Phenotypic characterization was also done by Ezy MIC strip for the confirmation of ESBL producers. Ceftazidime and ceftazidime + clavulanic acid containing strip was used. Out of the total samples screened for ESBL production only 84 samples depicted positive results by Ezy MIC strip (Table-4). The phenotypic characterization by CDDT method revealed most of the samples were resistant to cefotaxime, cefpodoxime and few were resistant toward ceftazidime these findings correlates with the European Union recommendations as ESBL producers are usually resistant to cefotaxime, variably resistant to ceftazidime, and susceptible to cefoxitin [16]. Thus, in reference to the current recommendations [5] cefotaxime was included as marker for present investigations. Resistance to ESBL-producing isolates testing with ceftazidime would improve the ability to identify the organism and also enhance the sensitivity to identify the isolates producing certain beta-lactamases belonging to SHV and TEM families of enzymes which are ceftazidimases and have much lower activity than cefotaximases. As per the recommendation of the European Union use of specific chromogenic medium avoided the identification of the colonies belonging to the other species within Enterobacteriaceae. Enrichment at this level with cephalosporins as stated in the scientific report of EFSA, influence the bacterial conjugation, exchange of resistance plasmids and increase the sensitivity of the method [5]. This correlates with our findings as initial screening gave 135 samples positive for ESBL *E. coli*, and all the samples showed positive results in the phenotypic characterization by CDDT method.

**Table 4:** Comparative sensitivity of methods of phenotypic characterization of ESBL *E. coli*.

Type of samples	CDDT method	DDST method	Ezy MIC strip
Positive samples	135	115	84
Negative samples	0	20	51
Total number of samples	135	135	135
Percent sensitivity	100	85	62
Chi-square value	67.7**		

** p<0.01 results are highly significant. ESBL=Extended spectrum beta-lactamase, *E. coli*=*Escherichia coli*, CDDT=Combined disc diffusion test, DDST=Double disc synergy test

## Conclusion

Animal was apparently healthy during the slaughter and was used for the human consumption; the prevalence of ESBL-producing *E. coli* which is a commensal bacteria indicating the problem of antibiotic resistance against beta-lactam antibiotic group which even includes third and fourth generation cephalosporins. CDDT method was found to be most sensitive among the three and most of the isolates were resistant toward cefotaxime discs and Ezy MIC strip method was the least the reason behind this was Ezy strip method is based on MIC values and the range of MIC could have been beyond the MIC range of the strip. As these organisms carry their genes on the plasmid due to horizontal gene transfer co- or multi-resistance against other antibiotics are also possible which could be an alarming sign specially for the poultry and human in contact with the birds.

## Authors’ Contributions

AS designed and planned this research work collected the samples and executed the entire work RKS and YPS guided and monitored the entire research work. NS contributed in the collection of samples and also helped in the designing of work plan VG and SJ analyzed the data and were involved in the experiment. All authors contributed equally in preparation and revision of the manuscript. All authors read and approved the final manuscript.
